# Effect of immobilization mask and beam energy on dose coverage to small joints in treating osteoarthritis with low‐dose radiation therapy

**DOI:** 10.1002/mp.18099

**Published:** 2025-09-03

**Authors:** George X. Ding, Kenneth L. Homann, Eric T. Shinohara

**Affiliations:** ^1^ Department of Radiation Oncology Vanderbilt University School of Medicine Nashville Tennessee USA

**Keywords:** dose accuracy for small joints, immobilization mask, low dose for osteoarthritis, Monte Carlo

## Abstract

**Purpose:**

Osteoarthritis (OA) is the most common form of arthritis, affecting over 32 million Americans. Low dose radiation therapy (LDRT) is being used to treat OA, including small joints. Treatment energies recommended include both orthovoltage and 6 MV photons. This study evaluates treatment plan accuracy of small joints using a commercial treatment planning system (TPS) when 6 MV is used. The effect of bolus and immobilization mask on target dose coverage and the use of 2.5 MV beams are also studied.

**Methods:**

Monte Carlo calculated dose distributions were used to evaluate the dose calculation accuracy of small joints by the Varian Eclipse system (AAA V.16) for one patient. The CT based dose calculations with‐ and without an Aquaplast immobilization mask using 6 MV and 2.5 MV beams were compared. The target dose coverages were analyzed using a dose volume histogram (DVH). The effect of the Aquaplast mask on target dose coverage was evaluated. The doses calculated by Monte Carlo (MC) were regarded as the Gold Standard.

**Results:**

The dose calculated by the Eclipse system significantly underestimated D_95_ target coverage by up to 21% of the prescribed dose. D_95_ was 92.9%, 91.7% and 89.6% of prescribed dose with 1 cm bolus, with a custom Aquaplast mask, and without a custom Aquaplast mask based on MC calculations, respectively, as compared to 86.8%, 83.2% and 73.9% when using Eclipse.

**Conclusion:**

Eclipse calculations are less accurate, and underestimate D_95_ target dose by 7% even with bolus. When Monte Carlo is not available, prescribing to the D50 in Eclipse can lead to an actual D_95_ coverage of >90%. The immobilization mask provides adequate buildup for 6 MV beam. To obtain the full benefit of lower‐energy beams the 2.5 MV‐flattened beam provided the best dose coverage regardless of the use of a mask when treating small joints.

## 1 INTRODUCTION

Radiotherapy (RT) generally uses high doses (>50 Gy) to treat malignant disease. However, low to intermediate doses (3–50 Gy) can provide effective control for a number of benign conditions, including inflammatory/proliferative disorders.^1^ Osteoarthritis (OA) is the most common form of arthritis, affecting over 32 million Americans. Low dose radiation therapy (LDRT) has been used to treat symptomatic OA.^2^, ^3^, ^4^ For treating small joints, treatment energies recommended include orthovoltage in the range of 100–200 kV or 6 MV photons. At our institution, OA is typically treated with 300 cGy in six fractions, given every other day. Patient specific CT based dose calculations are performed using the Varian Eclipse (V.16) treatment planning system (TPS). Although the TPS passed rigorous tests prior to clinical use, the dose calculation uncertainty is not well known when applied to the treatment of small joints. The benefits of bolus in such scenarios are also not clear. This study aims to determine if a commercial TPS can accurately calculate dose coverage of small joints when 6 MV photons are used and the effect of an Aquaplast immobilization mask on target dose coverage. In addition, we explore the benefits of using a lower energy 2.5 MV beam, which may provide better surface coverage of joints due to less need for buildup. While the benefit of 2.5 MV photons in SRS application^5^, ^6^ is limited by its low dose rate (60 MU/min), this is not an issue in LDRT application where a dose of 50 cGy can be delivered in less than a minute.

**NOVELTY**

Using Monte Carlo simulations, this study evaluated treatment plan accuracy of small joints by a commercial treatment planning system, Eclipse, when 6 MV was used. It also explores the effect of bolus, immobilization mask on target dose coverage. The study also investigates the advantage of using a native 2.5 MV imaging beam and a flattener added 2.5 MV therapeutic beam. The study showed that the Eclipse calculated plan underestimated D_95_ target dose by 21%. Even with 1 cm bolus it underestimated by 7%. The study also provided a way to reduce the error in Eclipse when Monte Carlo was not available. The study found that the immobilization mask provides adequate buildup for 6 MV beam. To obtain the full benefit of lower‐energy beams the 2.5 MV‐Flattened beam provided the best dose coverage regardless of the use of a mask when treating small joints.

## 2 METHODS AND MATERIALS

The Monte Carlo simulation technique is regarded as a gold standard for dose calculations in a wide range of beams used in radiotherapy.^7^ The EGSnrc system, which includes the user codes, BEAMnrc^8^ and DOSXYZnrc, were developed for radiotherapy use and have been widely used for therapy and imaging purposes. The EGSnrc system was used to generate the therapeutic 6 MV^9^ and a 2.5 MV^10^ beams that are currently utilized for imaging by Varian. The accuracy of simulated beams has been validated in previous studies.^9^, ^10^ The simulated beams were stored in phase‐space files^8^ and used to calculate dose distributions.

**FIGURE 1:**
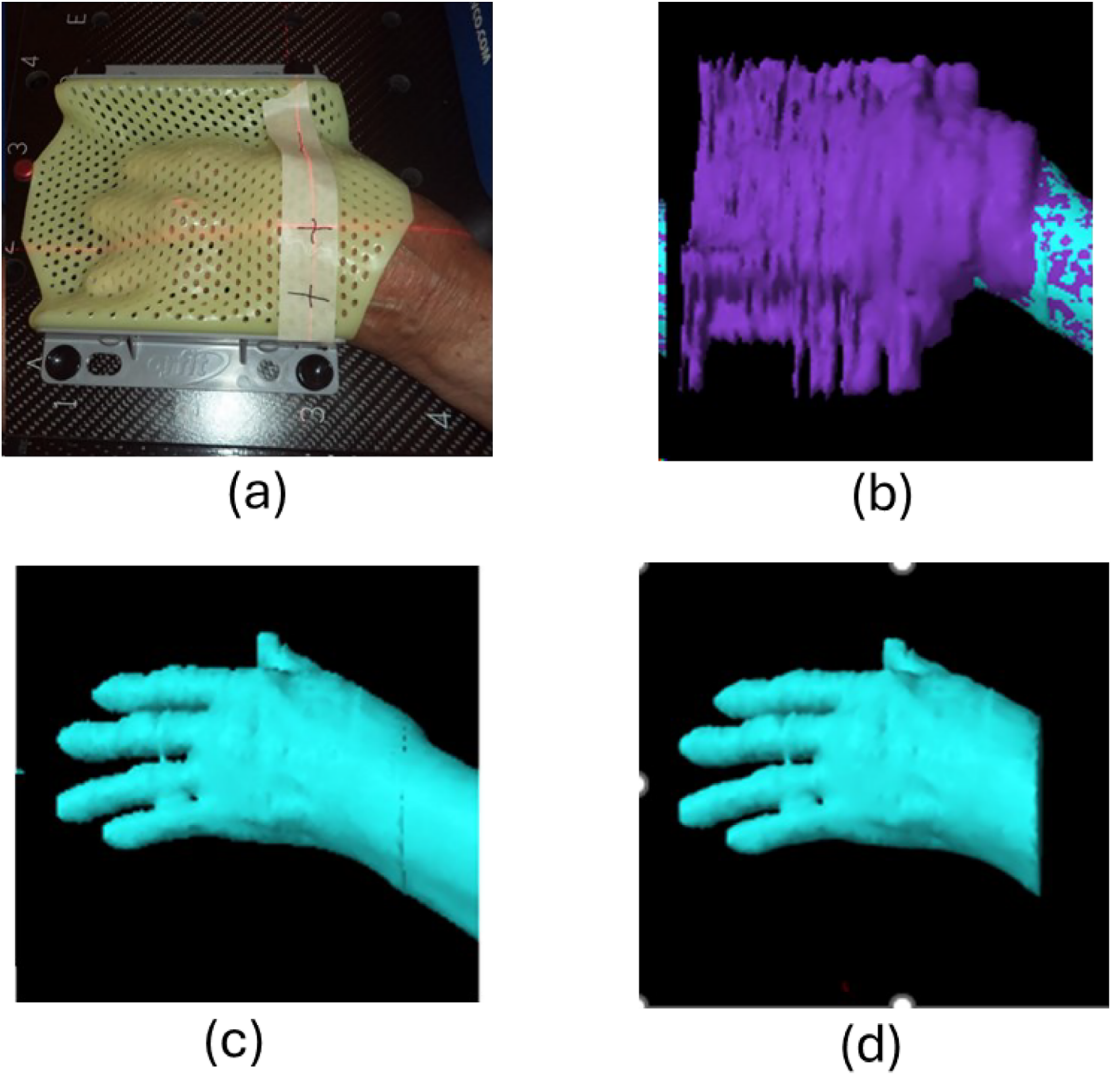
(a) photo of patient left hand CT Sim setup, (b) contour with mask, (c) left hand contour, and (d) target contour, which is in the primary beams.

This study used a previously treated patient plan for OA of the hand and compared the calculated dose distributions between Eclipse and Monte Carlo. Figure 1 shows a (a) setup photo of the patient's left hand with Aquaplast immobilization mask from CT based treatment planning simulation, (b) mask and hand contour volume for Eclipse dose calculations, (c) left hand contour, and (d) target contour for dose coverage evaluations. The left‐hand contour is at the skin surface while the target contour only includes the part that is in the primary beams.

The previous plan used 6 MV beams to treat a left hand with parallel opposed AP/PA beams of a field size of 20 x 18 cm^2^. The CT based dose calculations with‐ and without an Aquaplast immobilization mask using 6 MV and 2.5 MV beams were compared. In the “without mask” scenario, the mask was set to air, while for “with mask”, the mask remained as scanned for a fair and accurate comparison. Doing so avoided any geometrical changes that a rescan of the hand without the mask might cause. The target dose coverage was evaluated using a dose volume histogram (DVH). In all cases, D_50_ was kept at 100% of the prescription for comparison purposes.

**FIGURE 2:**
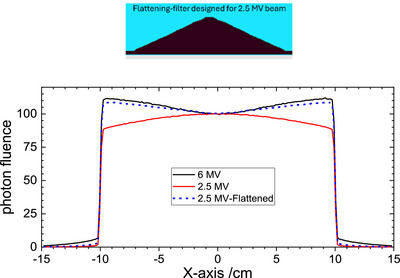
Upper graph: The design of the cone shaped flattening‐filter for 2.5 MV beam (not to the scale); Lower graph: the photon fluence profiles at SSD = 100 cm as a function of off‐axis for 6 MV (black line), 2.5 MV (red line) and 2.5MV‐Flattened (blue‐dash line) beams, respectively.

The 2.5 MV beam is designed for imaging and its beam profiles are peaked at the center as typical with a flattening‐filter‐free (FFF) beam. For a fair comparison in evaluating the benefit of a low energy beam, we flattened the 2.5 MV beam by adding a flattening‐filter. We designed the flattener by modifying previous Varian Clinac low‐energy beam flattening‐filters to achieve a similar flatness to beams used for therapy. The shape of the flattening‐filter used for the 2.5 MV beam is shown in Figure 2. The material used for the 2.5 MV beam flattener is titanium, which is commonly used for beam exit windows in the linac head.

## 3 RESULTS

Figure 2 shows a comparison of photon beam fluence profiles of a 6 MV, a 2.5 MV, and a 2.5 MV‐flattened beam, respectively.

Figure 3 shows the dose distributions as a color‐wash calculated by Eclipse and Monte Carlo, respectively. It is worth noting that in the Eclipse dose calculations the body contour started a few centimeters above the mask to increase the accuracy of the Eclipse calculations.^11^ The areas covered by a minimum dose of 100%, 95%, 90%, 85% and 50% are shown in different colors.

FIGURE 3Isodose distributions using 6 MV beams (a) with 1 cm bolus and mask; (b) with mask; (c) without mask in color‐wash shown in axial, coronal, and sagittal planes for Eclipse and Monte Carlo calculations respectively; (d) and (e) Monte Carlo calculated isodose distributions using 2.5 MV and 2,5 MV‐Flattened beams with and without mask, respectively.

**Figure:**
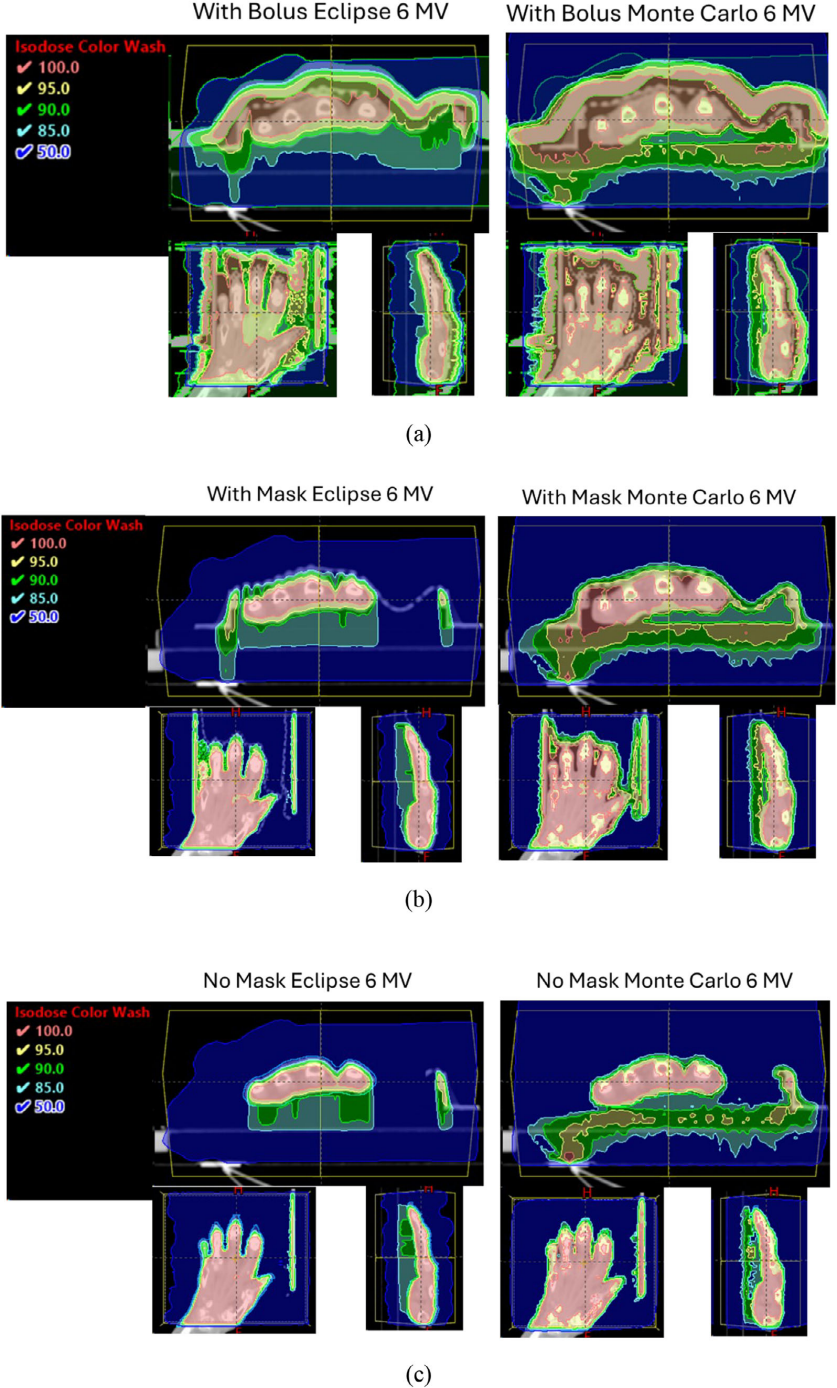

**Figure:**
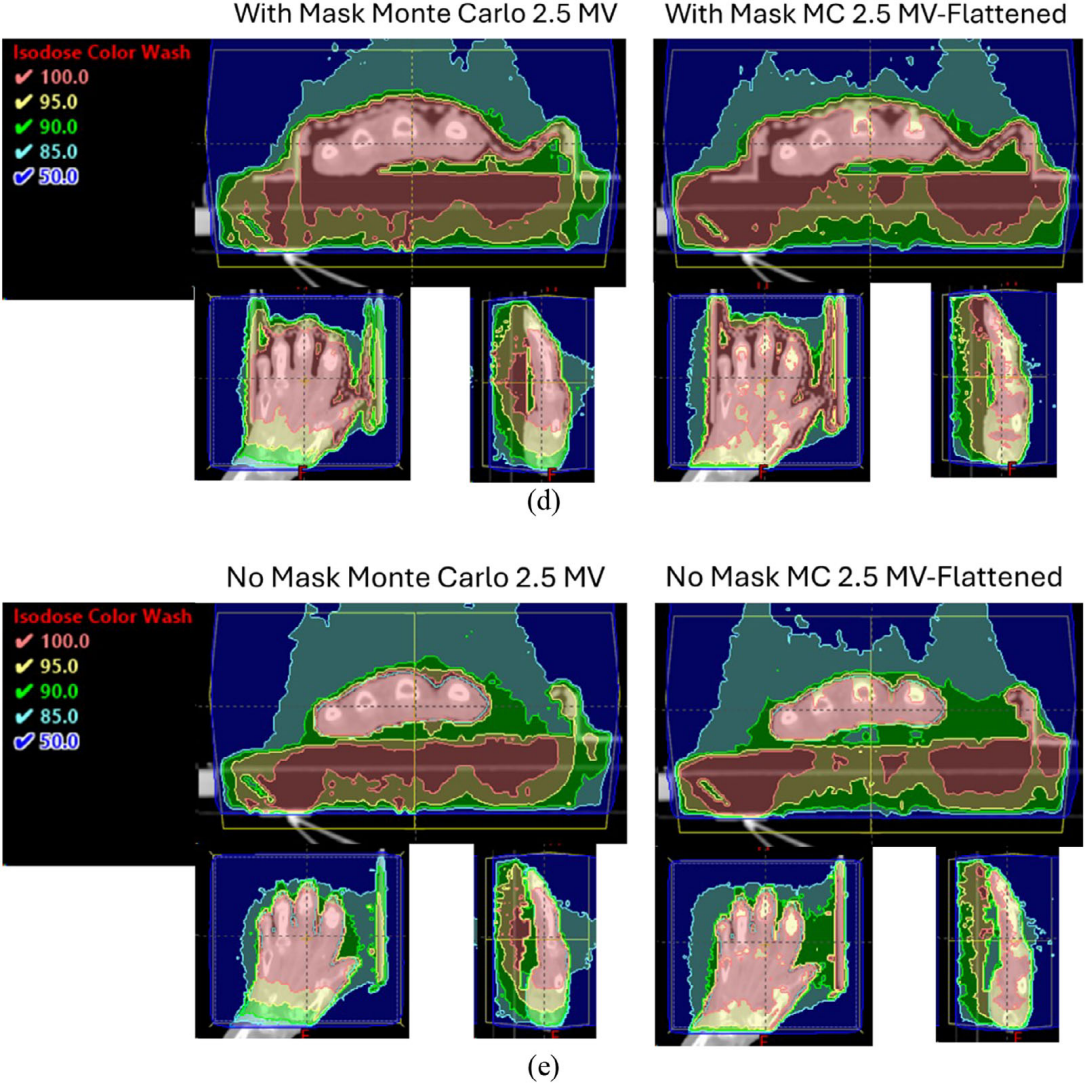

**FIGURE 4:**
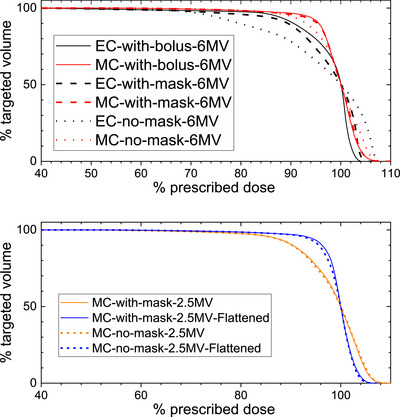
Comparison of DVH plots for each treatment scenario when using Eclipse Calculations (EC) and Monte Carlo (MC) calculations.

Figure 4 shows DVH curves for each scenario. In this study, the Monte Carlo calculation was regarded as the Gold‐Standard. The statistical uncertainty in this study is kept <1% for calculated dose voxels in the target volume in all Monte Carlo calculations. The target dose coverages were analyzed using a DVH. Much larger target dose coverage differences were observed between with and without mask in Eclipse dose calculations while there were smaller differences seen in the corresponding dose distributions calculated using Monte Carlo techniques. It is known that model‐based dose calculation algorithms are less accurate in inhomogeneous media, especially at the interface. In the case of treating small joints, using Eclipse (AAA V.16) dose calculations are not as accurate, even with 1 cm bolus added on top of the mask. The improvements for target dose coverage with 2.5 MV‐Flattened beam are noticeable.

Table 1 presents dose coverage details based on DVH analysis. The doses calculated by Eclipse significantly underestimated target coverage by 7%–21% of prescribed dose when treating small joints even with 1 cm added bolus on top of the mask. D_95_ was 92.9%, 91.7% and 89.6% of prescribed dose with 1 cm bolus, with a custom Aquaplast mask, and without a custom Aquaplast mask based on MC calculations, respectively, as compared to 86.8%, 83.2% and 73.9% when using Eclipse. The custom immobilization mask provides nearly adequate buildup for 6 MV beam when treating small joints. The target dose coverage was improved when a 2.5 MV beam was used when the beam was flattened, regardless of whether a mask was used or not.

**TABLE 1:** List of calculated minimums, mean, and maximum percentage of prescribed doses to the target and the minimum prescribed dose received by 100% (D_100_), 95% (D_95_), 90% (D_90_), 50% (D_50_), and 10% (D_10_) of the target volume based on DVH from Eclipse (EC) and Monte Carlo (MC) systems with and without hand mask.

Beam	System	Mask	Min	Mean	Max	D_100_	D_95_	D_90_	D_50_	D_10_
6 MV	EC	Without	28.7	96.4	108.1	28.7	73.9 (21%)	79.7	100	106.3
6 MV	EC	With	27.8	97.3	105.9	28.4	83.2 (10%)	89.4	100	103.5
6 MV	EC	+Bolus	34.4	97.4	104.5	34.5	86.8 (7%)	90.5	100	102.0
6 MV	MC	Without	52.0	99.2	111.0	52.4	89.6	93.0	100	104.9
6 MV	MC	With	51.0	99.2	111.1	51.6	91.7	95.3	100	103.6
6 MV	MC	+Bolus	50.3	99.2	109.4	50.9	92.9	95.6	100	103.5
2.5 MV	MC	Without	45.2	98.3	110.3	45.7	86.3	90.2	100	105.0
2.5 MV	MC	With	45.7	98.3	110.5	45.7	86.4	90.3	100	104.8
2.5 MV‐Flattened	MC	Without	50.5	99.1	107.7	50.8	93.0	95.6	100	102.8
2.5 MV‐Flattened	MC	With	50.7	99.3	107.6	51.0	93.7	96.2	100	103.0

*Note*: The values shown in brackets () in column D95 indicate percentage errors of Eclipse calculations.

## 4 DISCUSSION

**FIGURE 5:**
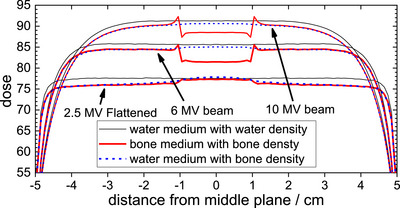
Comparison of differences between Monte Carlo calculated dose‐to‐medium and dose‐to‐water for 2.5 MV‐flattened, 6 and 10 MV beams. Shown are parallel opposed beams incident on slab phantoms from left to right, respectively, with iso center at the middle of the phantom. The phantom slabs consist of two 4 cm‐thick water slabs (from −5 cm to −1 and 1–5 cm) and one 2 cm bone slab (from −1 to 1 cm). The black solid line is calculated by assigning the bone slab to water medium with water density, the red dot line calculated by assigning the bone slab to bone medium with bone density, and the blue dash line is calculated by assigning the bone slab to water medium with bone density.

While the dose used to treat OA is low and the risks of retreatment are modest, optimizing dose coverage remains important. Dose calculation algorithms, such as AAA implemented in the Eclipse TPS, have limitations in predicting dose accurately, especially at interface and buildup regions. A bolus creates buildup depth, which can effectively avoid these limitations. However, the bolus also leads to the skin receiving the full dose. It is also worth noting that Monte Carlo calculated doses are dose‐to‐medium while the Eclipse calculated doses are dose‐to‐water. For soft tissues, the difference is negligible. However, dose‐to‐bone for photon beams is lower than dose‐to‐water depending on the beam energy. Figure 5 shows differences between Monte Carlo calculated dose‐to‐bone and dose‐to‐water for 2.5 MV, 6 MV and 10 MV photons for parallel opposed beams with 10x10 cm^2^ field size incident on a slab composed of water and bone phantoms. The curve referred to as “water medium with water density” was calculated by assigning the bone slab to water medium with water density. The curve referred to as “bone medium with bone density” was calculated by assigning the bone slab to bone medium with bone density, and the curve referred to “water medium with bone density” was calculated by assigning the bone slab to water medium with bone density. The calculated differences between “bone medium with bone density” and “water medium with bone density” are the differences between dose‐to‐bone and dose‐to‐water. It is seen that dose‐to‐bone is 0.7%, 4.3% and 2.4% lower than dose‐to‐water for 2.5 MV‐Flattened, 6 MV and 10 MV beams, respectively. For 15 MV beam (not show), the dose‐to‐bone is only 1.5% lower than dose‐to‐water. For the 2.5 MV imaging beam (not shown), the dose‐to‐bone is 1.1% higher than dose‐to‐water due to its unfiltered low‐energy photons. The effect of higher dose‐to‐bone for 2.5 MV beam is clearly visible in Figure 3d and 3e where the small bone joints are completely covered by 100% isodose line in axial slice. The calculated curves were normalized to the calculated values of the dose‐maximum of corresponding beam energy at central‐axis with a 10x10 cm^2^ field incident on water phantom with SSD = 95 cm. The largest difference (4.3%) between dose‐to‐bone and dose‐to‐water occurs at photon beam energies of 6 MV beam has some clinical relevance in treating bone joints.

This difference between dose‐to‐water and dose‐to‐medium calculations is clearly visible in Figure 3, where the Monte Carlo calculated bone dose is lower than surrounding soft tissue. Monte Carlo calculated dose‐to‐medium is the most accurate representation of absorbed dose to bone. How then can we best apply these findings clinically when we do not have Monte Carlo calculations available for these specific patients? Review of DVH comparisons shown in Figure 4 and Table 1 between Eclipse and Monte Carlo for 6 MV photons suggests that prescribing to the D_50_ in Eclipse leads to an actual D_95_ coverage of > 90% for Monte Carlo calculations.

Additionally, adding a flattener to the native 2.5 MV imaging beam provided the full benefits of low‐energy therapeutic beams. The overall DVH for 2.5 MV‐Flattened beams provided superior coverage with a lower hot spot. In addition, the 2.5 MV or 2.5 MV‐Flattened beams showed little deviation in DVH structure with or without the mask present. It is worth noting that the dose rate is reduced by 35% with the flattener, which only marginally slows down the treatment. The benefit of using the 2.5 MV‐Flattened beam seen in this study for LDRT may encourage the manufacturer to include a flattened 2.5 MV beam, which is very feasible.

## 5 CONCLUSION

We evaluated the accuracy of dose calculations by the Varian Eclipse treatment planning system when treating small joints. Model‐based Eclipse (AAA V.16) calculations are less accurate when calculating dose to small joints, and it significantly underestimated D_95_ target coverage by up to 21% of prescribed dose depending on use of bolus, mask, or nothing. Even with 1 cm bolus added on top of the mask, Eclipse still underestimated D_95_ by 7%.

When Monte Carlo is not available, prescribing to the D_50_ in Eclipse can lead to an actual D_95_ coverage of > 90% based on results from this study. When using 6 MV photons, Monte Carlo calculations showed that the immobilization mask has a buildup effect which may eliminate the need for additional bolus. To explore the full benefit of using lower‐energy 2.5 MV beams in LDRT for symptomatic OA, we modified the native 2.5 MV imaging beam by adding a flattener and produced a 2.5 MV‐Flattened beam. The 2.5 MV‐Flattened beam provided the best dose coverage regardless of the use of a mask when treating small joints due to the reduction of the Rx dose to tissue anterior to the bone and joint intended to be treated. The use of a 2.5 MV flattened beam with mask may provide the best coverage of small joints without the need for additional bolus or normalizing to an isodose curve of < 100%.

Because the primary radiation targets are the bone joints in this investigation, we also investigated the differences between dose‐to‐medium and dose‐to‐water. This study found that the differences between dose‐to‐bone and dose‐to‐water vary and fluctuate as a function of photon beam energies with the largest difference occurring at 6 MV. At low‐beam energy, such as 2.5 MV imaging beam, the dose‐to‐bone is 1.1% higher than dose‐to‐water, while for a 2.5 MV‐Flattened beam the dose‐to‐bone is 0.7% lower than dose‐to‐water. The higher dose‐to‐bone for 2.5 MV beam may be beneficial since primary targets are bone joints. For 6 MV, 10 MV and 15 MV beams, the dose‐to‐bone is 4.3%, 2.4% and 1.5% lower than dose‐to‐water, respectively. These additional findings may have some clinical relevance in treating bone joints.

## CONFLICT OF INTEREST STATEMENT

The authors have no conflicts to disclose.
